# Current Stem Cell Delivery Methods for Myocardial Repair

**DOI:** 10.1155/2013/547902

**Published:** 2012-12-27

**Authors:** Calvin C. Sheng, Li Zhou, Jijun Hao

**Affiliations:** ^1^School of Medicine, Vanderbilt University, 2220 Pierce Avenue, Nashville, TN 37232, USA; ^2^College of Veterinary Medicine, Western University of Health Sciences, 309 East Second Street, Pomona, CA 91766, USA

## Abstract

Heart failure commonly results from an irreparable damage due to cardiovascular diseases (CVDs), the leading cause of morbidity and mortality in the United States. In recent years, the rapid advancements in stem cell research have garnered much praise for paving the way to novel therapies in reversing myocardial injuries. Cell types currently investigated for cellular delivery include embryonic stem cells (ESCs), induced pluripotent stem cells (iPSCs), and adult stem cell lineages such as skeletal myoblasts, bone-marrow-derived stem cells (BMSCs), mesenchymal stem cells (MSCs), and cardiac stem cells (CSCs). To engraft these cells into patients' damaged myocardium, a variety of approaches (intramyocardial, transendocardial, transcoronary, venous, intravenous, intracoronary artery and retrograde venous administrations and bioengineered tissue transplantation) have been developed and explored. In this paper, we will discuss the pros and cons of these delivery modalities, the current state of their therapeutic potentials, and a multifaceted evaluation of their reported clinical feasibility, safety, and efficacy. While the issues of optimal delivery approach, the best progenitor stem cell type, the most effective dose, and timing of administration remain to be addressed, we are highly optimistic that stem cell therapy will provide a clinically viable option for myocardial regeneration.

## 1. Introduction

Cardiovascular diseases (CVDs) are the number one cause of mortality worldwide, and their prevalence is projected to remain the single leading cause of death [1]. Two major types of CVDs, hypertension and coronary artery disease, can lead to myocardial infarction (MI) characterized by death of cardiomyocytes and eventual heart failure [2]. Despite rapid advancements in pharmacologic and surgical approaches over the last several decades, heart failure still remains one of the significant diseases with unresolved dilemmas. So far, the only definitive treatment for heart failure is heart transplantation, which is precluded from wider application due to the limited availability of donor hearts and complications from immunosuppressive therapies [3]. Therefore, there are great clinical interests to pursue novel treatments for improving heart function. 

In recent years, stem-cell-based therapy has attracted much attention as a viable approach to treating heart failure. Increasing studies have indicated that stem-cell-based cardiovascular regeneration has the potential to revolutionize current treatments for patients with ischemic heart disease [4, 5]. Despite the excitement surrounding stem-cell-based cardiac repair, many challenges still remain, such as validating the efficacy and robustness of various delivery methods. In this paper, we assess the current state of stem cell therapy in clinical application, explore the main strategies of cell delivery, and discuss the future direction of the field. 

## 2. Types of Stem Cells Used in Therapy

**Figure 1:**
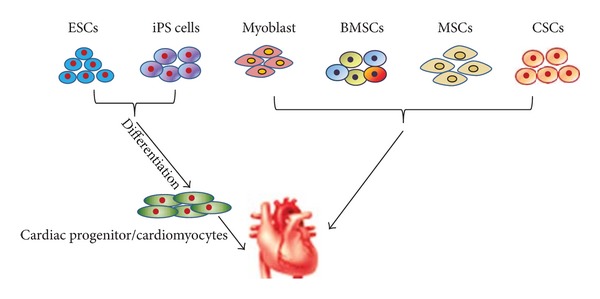
Types of stem cells used for cardiac regenerative therapy. Pluripotent stem cells including ESCs and iPS cells are generally differentiated to cardiac progenitor/cardiomyocytes which are then utilized for heart repair. In contrast, multipotent/unipotent stem cells, such as myoblast, BMSCs, MSCs, and CSCs, are generally used to restore heart function directly.

Pluripotent stem cells and multipotent/unipotent stem cells have been extensively studied for cardiac repair in experimental models and in clinical trials (Figure 1). Pluripotent stem cells, like embryonic stem (ES) cells and induced pluripotent stem (iPS) cells, are capable of differentiating into all cell types of the body including cardiomyocytes. In contrast, multipotent/unipotent stem cells can only differentiate into a limited number of cell types. In this section, we will briefly summarize and discuss the use of pluripotent and multipotent/unipotent stem cells for cardiac repair.

### 2.1. Pluripotent Stem Cells

#### 2.1.1. ES Cells

ES cells isolated from the inner cell mass of blastocysts are pluripotent and hold great promise as a source of cells for regenerative therapies in treating heart failure. In the past decade, significant progress has been made in efficiently differentiating ES cells into cardiac cells *in vitro* and engrafting the ES cell-derived cardiac cells into injured hearts for *in vivo* repair (review articles [6–8]). For instance, we have developed efficient chemical approaches to induce mouse ES cell cardiomyogenesis by timely modulating the BMP and Wnt/*β*-catenin signaling pathways [9, 10]. However, cardiomyogenesis of human ES cells is more challenging. One early study reported that coculturing human ES cells with mouse visceral endoderm-like (END-2) cells induced ventricular-like cardiomyocytes, though inefficiently [6]. Since then, several new methods have endeavored to more effectively promote cardiac formation from human ES cells by stage-specific activation and inhibition of various signaling pathways implicated in cardiac development of the early embryo [11–19]. In animal models, the transplantation of ES cell-derived cardiac cells into ischemic regions has shown improvements in myocardial performance [14, 20–26]. Nevertheless, the future clinical translation of pluripotent human ES cells faces many challenges including a lack of efficient approaches for human ES cell cardiomyogenesis, serious concerns of developing teratomas, immune rejection, and implicated ethical issues. 

#### 2.1.2. iPS Cells

The emergence of iPS cell technology has boosted tremendous enthusiasm for regenerative medicine as these cells reprogrammed from a patient's own somatic cells could, in principle, circumvent the ethical concerns and problem of immune rejection associated with human ES cells [27–32]. iPS cells are functionally equivalent to ES cells in their ability to differentiate into all types of body cells including cardiomyocytes. Thus far, a variety of methods have been developed to promote human iPS cell cardiac induction *in vitro* [15, 17, 33, 34]. Recently engrafting iPS cells or iPS cell-derived cardiomyocytes into ischemic hearts was shown to improve heart performance in animal models [33, 35]. However, clinical applications of iPS cells face several major hurdles such as low cellular reprogramming efficiency, epigenetic memory, oncogenic risks, low efficiency of cardiomyogenesis, and cell line to line variations [28, 29, 33, 36–38]. 

Recently, several groups have successfully transdifferentiated fibroblasts into cardiomyocytes. Srivastava and colleagues showed that postnatal cardiac or dermal fibroblasts can be switched to cardiomyocyte-like cells with combination of three transcription factors, Gata4, Mef2c, and Tbx5 *in vitro* [39]. The same group has further demonstrated that local delivery of these three transcription factors in murine hearts with coronary ligation can lead to formation of new cardiomyocyte-like cells and a decrease in infarct size [40]. Similarly, Olson's group further validated these reports by showing that fibroblasts can also be trans-differentiated to cardiomyocytes *in vitro *and *in vivo *by adding an additional transcription factor Hand2 to the three previously described [40].

### 2.2. Multipotent/Unipotent Stem Cells

Multipotent/unipotent stem cells, which are capable of giving rise of multiple or a single cell lineage(s), can be isolated from adult tissues or organs (Figure 1). The most studied multipotent/unipotent stem cells for heart repair are skeletal myoblasts, bone marrow stem cells, mesenchymal stem cells, and residential cardiac stem cells. Over the years, beneficial effects of multipotent stem cells on restoring the damaged heart functions have been extensively evaluated [41–48]. 

#### 2.2.1. Skeletal Myoblasts (Autologous)

Unipotent skeletal myoblasts are precursor cells of human skeletal muscle. These cells normally lie in a quiescent state but can reenter the cell cycle to proliferate and differentiate into functional skeletal muscle in response to injury [49]. The unique features that make skeletal myoblasts suitable for cardiac repair are their autologous origin, high proliferative potential, resistance to ischemia, and low risk of tumorigenesis [50, 51].

In extensive animal studies, skeletal myoblasts have been demonstrated to successfully engraft into the damaged heart areas, prevent left ventricular remodeling, and improve regional and global left ventricular function (review article [4]). These promising animal studies prompted early phase I clinical trials of skeletal myoblasts, which demonstrated the feasibility of surgical and catheter-based grafts [52–55]. While multiple phase I results showed promising results, Menasché and colleagues continued their assessment in a phase II study (NCT00102128) and found that the myoblast autologous grafts failed to significantly improve cardiac function as initially hoped [56]. The efficacy of the transplant seemed to fluctuate depending on graft volumes, which suggests that its viability in the future rests on resolving several key issues such as cell retention and postengraftment cell survival [57]. 

#### 2.2.2. Bone-Marrow-Derived Stem Cells (Autologous)

Bone marrow stem cells (BMSCs) are among the best described multipotent stem cells for transplantation because they are easily accessible, readily propagated in culture, and do not require adjunctive immunosuppressive therapy. A systematic review of 33 randomized clinical trials (*n* = 1765) demonstrated that patients treated with BMSC therapy for acute MI did not demonstrate a statistically significant difference in morbidity or mortality [58]. However, moderate improvement in LVEF was noted in the short term and was maintained between 12 and 61 months [58, 59]. The beneficial effects of BMSCs on heart repair have been hypothesized to be due to paracrine signaling [60, 61], but the exact mechanism by which BMSCs exert their action for cardiac improvement remains to be determined. 

Hematopoietic stem cells (HSCs) are one subset of BMSCs that have been shown in the COMPARE-AMI trial to improve LV function, but it is unclear whether this is due to cell differentiation into cardiomyocytes [62–64]. Transplantation of endothelial progenitor cells (EPCs), another subset of BMSCs, has also been shown to improve heart function in a slightly different manner [65, 66]. The EPCs do not differentiate into cardiomyocytes, but their ability to differentiate into endothelial cells leads to improved angiogenesis, thus increasing the delivery of oxygen and nutrients to host cardiomyocytes and endogenous stem cells [67].

#### 2.2.3. Mesenchymal Stem Cells (Autologous)

Mesenchymal stem cells (MSCs) are another well-described group of cells used for cardiac transplantation. These cells can be derived from a variety of different tissues like cord blood, BM, and adipose tissue. The MSCs can differentiate into various mesenchymal lineages such as skeletal myoblasts, chondrocytes, adipose tissue, and cardiomyocytes *in vitro* [68–70]. The major advantages of MSC-based cell therapy for cardiac repair lie in their ability to promote growth, survival, or differentiation of other cells in the infarction area by paracrine secretion of cytokines as well as their immunosuppressive effect [71, 72]. However, beneficial effects of MSCs on restoring the damaged heart function are often limited and transient, and there is still little evidence that adult stem cells can differentiate into myocardial cells [42–45, 47, 73]. 

#### 2.2.4. Resident Cardiac Stem Cells (Autologous)

One of the exciting new developments in the field of cardiac regenerative therapy is the identification of cardiac stem cells (CSCs) and their use in clinical trials [74–77]. These cells express receptor tyrosine kinase c-kit on the surface and are capable of differentiating into three major cardiac lineages (myocytes, endothelial cells, and vascular smooth muscle cells). Recently, two phase I studies (NCT00474461 and NCT00893360) have independently observed promising improvements in ventricular function through intracoronary infusions of CSCs [78, 79]. In the SCIPIO study (NCT00474461), Bolli et al. reported their preliminary findings on patients with postinfarction LVEF <40% before coronary artery bypass grafting (CABG) [78]. The patients receiving the CSC infusion had reduced infarct sizes with improvement in LV systolic function. Similarly, in the CADUCEUS trial, Makkar et al. reported improved regional contractility and reduced postinfarction scar size at six months after cell infusion [79]. While the initial clinical result of CSCs is exciting, caution should be taken as the study is still ongoing, and long-term assessment of larger randomized control trials is still needed. 

## 3. Methods of Stem Cell Delivery

Within the past decade, the potential of stem cell therapy has generated much excitement and led to significant progress in ultimately paving the way to clinical use. Enormous work has gone into identifying and characterizing the best approach to stem cell delivery. In the following, we will discuss the available modalities currently adapted in clinical studies. 

### 3.1. Direct Surgical Intramyocardial (IM) Injection

This epicardial procedure has been the most direct, precise, and accurate approach for injecting stem cells to an infarcted region of the heart. The location can be identified preoperatively using echocardiography and nuclear imaging and, during surgery, by empirical observation [80]. Direct intramyocardial injection can be typically done either during thoracotomies for open-heart surgeries like CABG [81] or as separate procedures performed without cardiac arrest via lateral minithoracotomies [82]. It offers a distinct advantage of targeting localized myocardium without perturbing surrounding tissue and vasculature. Thereby, it circumvents the need to address complex issues such as mobilization and homing of the transplanted cells. 

The biggest drawback when comparing with other delivery systems is the invasive nature of the operation. There are greater risks for complications and mortality including potential myocardial perforation at the site of injection, systemic embolization, and cardiac arrhythmias as with all forms of endocardial injection [83]. In a swine study, leakage from the injection site was also observed during and immediately after the procedure, yielding lesser total cell retention when compared with the catheter-based endomyocardial approach which will be described in the following [84]. Recovery period is also substantially prolonged. For these reasons, it would also not be very feasible for repeated use of this delivery method on the same individual.

Since Hamano et al. described the first successful bone marrow stem cell transplantation during CABG in 2001 [85], 13 additional studies have been evaluated for recovery of cardiac function (Table 1) (reviews articles [86, 87]), and the majority of which showed promising evidence for improvement of the left ventricular function. Contrary to the current indications, results from a recently completed phase III study (NCT00462774) indicate that surgical injection of bone marrow stem cells had no relevant effects on left ventricular function and heart failure symptoms, thereby unraveling nearly 10 years of work since the inception of its own pilot trials [88]. Meanwhile, another phase III trial (NCT00950274) currently underway will serve to either further validate or refute the negative findings of NCT00462774 by also testing the functional benefit of BMSC surgical injections during CABG. 

### 3.2. Catheter-Based Intramyocardial (IM) Administration

Percutaneous intramyocardial injection is more commonly performed in patients with chronic heart failure secondary to ischemic heart disease to avoid dealing with obstructed coronary arteries all together [106]. There are currently two delivery methods available: (1) transcoronary venous approach and (2) transendocardial approach. Unlike the surgical approach, catheter-based intramyocardial injection can be extended to surgically high-risk patients and be repeated if necessary because of its less invasive nature. One of the main questions currently under investigation is identifying the most effective catheter. There are five of such devices being tested in clinical trials for cell and gene therapies (Helix, MyoCath, Myostar, Stiletto, and TransAccess Delivery System). They differ in specific design aspects and materials used based on the anatomic approach, the first four in a transendocardial manner while the latter via an epicardial transcoronary venous method. In summary, MyoCath and Myostar catheters utilize an integrated system, meaning that the core and support catheters are combined into a single unit, whereas Helix and Stiletto core and support catheters are separate units [107]. 

In conjunction, another important aspect of catheter-based intramyocardial injections of cells is to optimize an imaging modality, either through two-dimensional (2D) or three-dimensional (3D) imaging. This type of procedure requires extensive imaging guidance within the ventricle to properly position the catheter at the site of injection. No longer limited by a 2D view via X-ray fluoroscopic guidance, the advancements in magnetic resonance imaging (MRI) now permit real-time 3D visualization of the entire procedure and the capability to anatomically distinguish between infarcted and healthy myocardium via contrast-enhanced imaging [108]. Several studies have demonstrated the feasibility of real-time MR-guided intramyocardial injections with the Stiletto system or a modified catheter [109–111]. 

Alternatively, the NOGA system (Biosense Webster, Diamond Bar, CA, USA) also provides real-time 3D color-coded endocardial imaging under the guidance of electromechanical mapping (EEM), an electromechanical sensor catheter that detects wall motion and electrical activity. In 1997, Gepstein et al. validated NOGA-based EEM as an accurate navigating system through *in vitro *and *in vivo* studies [112]. The promising animal study has led to numerous clinical pilot studies for cellular [113–115] and gene therapy [116, 117]. As with real-time MRI, this technique detects flux in magnetic fields instead of fluorescence, avoiding the long X-ray exposures, while still adequately identifying infarcted regions of myocardium [118, 119]. However, by comparison, this procedure itself is much lengthier, and the mapping requires extensive technical training. 

#### 3.2.1. Transendocardial Injection

With the introduction of new catheters and imaging modalities, physician scientists are now able to implement intramyocardial injections as a potential cellular therapy. The first of the two catheter-based techniques is the transendocardial approach that directly delivers cells through catheter-based EEM. This method was first implemented and validated in a swine model by Fuchs et al. who demonstrated improved cardiac function [120]. Since then, many clinical studies have been published with largely positive indications of efficacy (Table 2) (review articles [54, 87, 103–105]). While EEM is now accepted as an accurate modality for identifying ischemic myocardium [118, 119], the optimal catheter has yet to be determined. Several such as Helix (NCT00507468; NCT01087996) and Myostar (NCT01392625; NCT00790764; NCT01076920) have been integrated with EEM and are currently being tested in clinical trials. Similar to other injection techniques, there is always a low risk for potential wall perforations and ventricular arrhythmia caused by either alterations in the gap junction orientation or release of inflammatory stimuli [121]. Two recent publications within the past year by Trachtenberg et al. (NCT00768066) and Perin et al. (NCT00203203) both reported positive results, with some indication of left ventricular functional improvement using Helix and Myostar, respectively. To date, neither has observed any significant adverse complications. With continuous and rapid advancements in catheterization techniques and imaging devices, transendocardial injection appears to be a very viable, feasible, and safe approach to further investigate in the future, pending the results of current clinical trials. 

#### 3.2.2. Transcoronary Venous Injection

The latter of the two catheter-based delivery systems is the transcoronary venous injection. This procedure was first evaluated by Thompson et al. using the TransAccess (TransVascular Inc., Menlo Park, CA) catheter in combination with an intravascular ultrasound (IVUS) imaging and demonstrated in a swine model its feasibility and safety [122]. A few years later, Siminiak et al. conducted the first phase I clinical trial in a small cohort (*n* = 10) yielding hopeful results. They confirmed the feasibility and safety of this procedure in human subjects [55]. Their six-month and twelve-month followups still showed no detectable arrhythmias, an overall improvement in ventricular function based on New York Heart Association classification, and six of the nine patients with an increase of between 3% to 8% ejection fraction [123]. Until the study is repeated with a larger sample population, it would be currently premature to draw a definitive conclusion regarding the transcoronary venous approach. This method via the venous system offers an alternative cell therapy for patients with occluded coronary arteries to consider. Some of the current limitations include difficulty delivering cells to the right coronary territory, and the variability in coronary veins from person to person makes the procedure extremely difficult. In contrast to the transendocardial approach, in which cells are injected perpendicularly into the left ventricular wall, the transcoronary venous approach allows parallel cell injection, which may result in greater cell retention.

### 3.3. Intravenous (IV) Infusion

Intravenous infusion is a selective technique only for treating post-AMI patients, as it is reliant on physiological homing signals to injured myocardium, a condition not present in chronic heart failure. The greatest advantage to this approach is its simplicity and least invasive delivery route, which opens the option of multiple intermittent infusion treatments. Its safety and feasibility have already been confirmed through a swine model study [124] as well as later in a phase I clinical study (NCT00114452) with promising results even during the 12-month followup [125]. There is currently a phase II study underway (NCT00877903) aimed at evaluating the efficacy of infusing *ex vivo* cultured adult human mesenchymal stem cells intravenously after AMI. 

While still a relatively new approach, some skeptics are concerned about the low delivery efficiency of *∼*0% reported by some studies [126–128]. The validity of these conclusions is questionable due to small cohorts (*n* ≤ 6) in these studies. But given that it is delivered through systemic circulation, there is an increased likelihood of infused cells becoming trapped in other organs, particularly the lungs, and eliminated by the reticuloendothelial system [129]. Specific cardiac homing and engraftment mechanisms should be better defined in order to obtain more consistent results. Until a larger-scale study like NCT00877903 reassesses the efficacy of intravenous infusion, it is difficult to conclude whether or not this approach will be a viable option, despite being feasible and safe. 

### 3.4. Intracoronary (IC) Artery Administration

Using standard balloon catheters, intracoronary infusion can directly deliver cells into myocardial regions via the coronary artery of interest. This procedure is the most clinically practiced form of cell delivery [106] and especially preferred following acute myocardial infarction, because it can be done simultaneously during a percutaneous coronary intervention for treating stenotic coronary arteries [130]. Stem cells are infused through the catheter in one of two manners: (1) nonocclusive angioplasty at slow or high flow rates while maintaining coronary flow or (2) stop-flow method by interrupting it with balloon occlusion [80]. The main advantages are its direct infusion into the target area and the resulting homogenous cell engraftment [131]. 

A inherent disadvantage is that it would be extremely difficult to deliver cells to areas not well perfused, in addition to the selective pressure of engrafted cells having to survive less-than-ideal nutrient-deprived and hypoxic conditions. New devices like the Cricket microinfusion catheters (Mercator, San Leandro, CA) are the first of their kind specifically designed for coronary perivascular delivery of cells, injecting through blood vessel walls into deeper tissue while causing minimal trauma with its microscopic puncture wound [132]. In a recent 2012 phase I study (NCT00677222), allogeneic bone-marrow-derived adherent adult stem cells were adventitially delivered into patients using Cricket microneedle catheters after AMI. Preliminary data shows improved ventricular function measured via ejection fraction and stroke volume 4 months later [133]. However, its efficacy needs to be fully evaluated in a larger sample size and followed over a longer duration. 

There also appears to be a threshold with regards to the size and dose of cells delivered using the intracoronary route before incurring possible embolization in the small coronary arteries and consequent vascular microinfarcts [106, 134]. Nonetheless, the technique has been proven to be relatively feasible and safe over its clinical use in the past decade. 

Looking at 30 clinical studies since 2002 (Table 3) (review articles [87, 159]), intracoronary infusion is still shrouded amidst controversy regarding its actual impact on the recovery of cardiac function. For instance, analysis of REPAIR-AMI (NCT00279175) in a 2-year followup shows mixed findings, with statistically nonsignificant difference in left ventricular ejection fraction but with significantly smaller infarct size and improved contractility between the experimental and placebo groups [160]. In the near future, completion of several phase III studies (NCT00765453, NCT101569178, NCT00279175, NCT00747708, and NCT01187654) and a 10-year evaluation after transplantation (NCT00962364) will help to elucidate whether or not intracoronary infusion may be the optimal method of delivery. 

### 3.5. Retrograde Coronary Venous (RCV) Delivery System

The final type of administration currently available delivers stem cells to the ischemic or infarcted region by advancing a single or double balloon catheter through the coronary sinus. The method is already ubiquitously used during cardiac surgery procedures for perfusing tissue with arterial blood or protective solutions as a prophylactic treatment against iatrogenic myocardial ischemia. It can be adapted as a stand-alone procedure, especially beneficial for patients with coronary artery obstructions and those unsuitable for CABG because the venous system is fully patent. Prior to cell infusion, a detailed anatomical map can be obtained by a coronary sinus venogram. In a swine model of myocardial injury, Vicario et al. in 2002 [161] and Yokoyama et al. in 2006 [162] both demonstrated that this method does not produce hemodynamical changes and reported observing autologous bone marrow stem cells in the myocardium and enhanced angiogenesis. A follow-up prospective clinical study in patients with chronic refractory angina by Vicario's group further confirmed its feasibility [163]. However, there are similar issues with RCV infusion as transcoronary venous injection, because they both utilize the venous system. While they avoid the arterial coronary obstructions, these procedures with the tortuosity of the venous system are difficult to navigate. As medical technology advances, these challenges can be overcome and results from preliminary studies above have already indicated clinically promising utility, warranting further investigation. 

### 3.6. Engineered Monolayer Tissue Transplantation

Engineered tissue transplantation is a novel solution to the poor cell engraftment and survival issue that has plagued most of the delivery methods previously discussed. This procedure aims to regenerate injured cardiomyocytes by providing a physical scaffold to enhance adherence. Many studies have been published regarding the efficacy of various biological-scaffolding constructs as an adjunct approach to improving stem cell delivery. In a recent review by Sui et al. in 2011, they reported findings on 5 natural materials (notably gelatin, Matrigel, and collagen) and 7 synthetic materials used for tissue engineering but ultimately concluded that the optimal scaffold has not been discovered [164]. June of this year, Li et al. bioengineered a novel thermosensitive and injectable hydrogel synthesized from four polymers, which they showed to have a >76% efficiency of inducing MSC differentiation into cardiomyocytes [165]. Furthermore, these cardiomyocytes were characterized as not only expressing cardiac markers but also developing calcium channels and gap junctions, suggesting great potential for regeneration of heart tissue in infarcted regions. Put into perspective, the current state of engineering myocardial tissue is still in its infancy, often leaving more questions than answers.

As the stem cell field advances, significant progress has also been made towards generating monolayer sheets of cells. This enables direct tissue transplants, preserving cell-to-cell adhesion within the monolayer of stem cells and minimizes loss of cell during the engraftment process [166]. In 2006, Miyahara et al. conducted one of the initial studies demonstrating this novel method on a mouse myocardial infarction model utilizing mesenchymal stem cells. This was a proof-of-concept, showing that the engrafted sheet onto the ischemic myocardium survived and gradually grew to form a thick stratum that included a mixture of newly formed vessels, undifferentiated cells, and some cardiomyocytes [167]. They showed that the sheet of cells acted through paracrine pathways to trigger angiogenesis, reversed wall thinning in the ischemic region, and improved cardiac function. A similar study by Bel et al. in 2010 investigated the effectiveness of a composite sheet construct of adipose tissue-derived stem cells and embryonic stem cell-derived cardiac progenitors transplanted into Rhesus monkeys and successfully demonstrated its safety in a mammalian model [166]. The promising results indicated the presence of newly differentiated cardiomyocytes and increased angiogenesis in the infarcted area. 

One step further, instead of transplanting a monolayer sheet of stem cells on a scaffold, it should theoretically be possible to graft a monolayer sheet of pure cardiomyocytes derived from induced pluripotent stem cells (iPSCs) or embryonic stem cells (ESCs). In 2007, Caspi et al. reported improved myocardial performance in infarcted rat hearts when human ESC-derived cardiomyocytes were transplanted [168]. The recent advent of iPSC-derived cardiomyocytes provides an autologous source of cells for engraftment. One of the main challenges with this will be to establish a robust protocol for generating a monolayer sheet of cardiomyocytes. To date, the vision of directly grafting a sheet of cardiomyocytes into a patient has not been proven feasible to be piloted in a clinical study due to the possibility of transplant rejection, teratoma formation, and arrhythmias, but the rapid advancements in the field leave much optimism for the future. 

## 4. Comparing Methods of Cell Delivery

To summarize, there are six broad approaches to cell delivery, each with its own unique aspect and subtle pros and cons. We believe that, instead of solely looking at the objective results based on cardiac function, the efficacy of these delivery systems should be evaluated on a case-by-case basis. In the recent decades, there has been an immense push across all fields of medicine towards a personalized medicine end goal. For instance, in a patient with a recent ischemic myocardial injury due to an obstructed coronary artery, intracoronary artery infusion may not be the optimal treatment route to consider, regardless of its reported experimental efficacy. When comparing these approaches to cell delivery, there are several main factors to consider.

One of the fundamental differences among the various stem cell transplantation procedures is the access route, whether it is through direct thoracotomy, coronary arteries, or venous system. While direct myocardial injection has been shown to be effective [169], there is still significant loss of transplanted cells due to myocardial contraction, leakage from the site of needle puncture, and venous washout [170]. Many patients seeking stem cell therapy have been diagnosed with chronic heart failure predominantly resulting from myocardial ischemia [171]. A common cause is coronary artery disease, which makes arterial access more difficult to maneuver. Like previously mentioned, the venous system effectively avoids that problem but at the cost of having to deal with tortuous and complex veins instead. Anatomically, arteries have relatively narrower lumens and transport blood under higher pressure than veins, increasing the difficulty of cell delivery and risk of perforations. One advantage is that arterial transplantation may show better engraftment results, because they are enriched with oxygen and other nutrients and they do not have valves, which may trap a proportion of the cells. Future investigations will be necessary to evaluate the efficacy differences of these treatment options. 

A more subtle distinction is between injection versus infusion. Injection usually refers to a more direct and localized delivery method by instrumental means, but not necessarily combining with anything around it. However, infusion is the addition of a substance into a solution, the bloodstream in the case of stem cell delivery, and interacting with the surrounding. When cells are delivered via infusion, they are more likely to diffuse throughout the entire circulatory system and are dependent on homing signals to target the injured myocardial region. If a better molecular targeting system can be elaborated, then infusions would be more viable than injections since these procedures are easier, safer, and better suited for multiple deliveries. In contrast, injections can be better guided via catheterization and imaging to a localized area of ischemic injury and then subsequently release the cells for engraftment. While the latter shows better improvement in left ventricle function [172], there is more potential for iatrogenic injuries like myocardial perforations and induced arrhythmia. 

Finally, in assessing all the available cell delivery methods, we should first consider the procedure from a bird's eye view. In a swine study assessing the efficacy of cell engraftment using surgical, IC, and coronary venous delivery systems, Hou et al. traced radioactively labeled transplanted cells and reported 11, 2.6, and 3.2% of them being retained, respectively [169]. Surgical intramyocardial is the most direct but also the most invasive, making the procedure extremely risky. Catheter-based intramyocardial approaches are feasible but limited by the scope and development of catheter technology and imaging modalities. Certain imaging like utilizing EEM requires extensive training. Intravenous, intracoronary artery, and retrograde coronary venous infusions are all subjected to the disadvantages discussed above. With regards to tissue engineering, the technology is still in its infancy and, while promising, will require much further inquiry to validate its therapeutic clinical potential for myocardial regeneration. 

## 5. Conclusion

In order to better understand the options presently available and ongoing research involved, we have conducted a comprehensive literature review of various cell delivery methods and evaluated them based on published clinical studies. The entire field of stem cell research is only decades old, so as we gain a better grasp of manipulating and utilizing stem cells, stem cell therapy will have tremendous therapeutic potential. Currently, several large yet elusive questions remain to be addressed: (1) optimal delivery approach, (2) best progenitor stem cell type, (3) most effective dose, and (4) timing of administration. Once we are able to resolve these obstacles, we can establish an optimized system for delivering cells with best cell engraftment and survival conditions. Concurrent work must be done using both animal models as well as in clinical trials to validate preliminary results. As many clinical studies show promising evidence and enter phase III trials, this is a particularly exciting time in the field, awaiting validation on a larger scale and creating the opportunity for stem cell therapy to be a routine procedure in the hospital one day. We strongly believe that, with the current trend and reported progress on cell delivery modalities, stem cell therapy will be a more robust and viable clinical treatment in the near future. 

## Figures and Tables

**Table 1:** Surgical direct myocardial injection studies.

Study	No. of patients	Cell type	No. of cells	Outcome
Hamano et al. (2001) [85]	5/0	ABMMNC	5 × 10^7^–1 × 10^8^	3/5 showed improvement in coronary perfusion
Patel et al. (2005) [81]	10/10	CD34 + BMC	2.2 × 10^7^	Significant improvement in cardiac function
Gavira et al. (2006) [89]	12/14	SMB	2.21 × 10^8^	Increased global and regional LVEF improvement in viability, and perfusion of cardiac tissue
Mocini et al. (2006) [90]	18/18	ABMMNC	2.92 × 10^8^	Improvement in LVEF and wall motion score index
Klein et al. (2007) [91]	10/0	CD133 + BMC	1.5 × 10^6^–9.7 × 10^6^	Improvement in LVEF
Ahmadi et al. (2007) [92]	18/9	CD133 + BMC	N/A	Improvement in wall motion score index and perfusion of cardiac tissue
Stamm et al. (2007) [93]	20/20	CD133 + BMC	5 × 10^6^	Improvement in LVEF and perfusion of cardiac tissue
Pompilio et al. (2008) [82]	5/0	CD133 + BMC	4 × 10^6^–12 × 10^6^	Improvement in perfusion but no significant improvement in LVEF
Zhao et al. (2008) [94]	18/18	ABMMNC	6.59 × 10^8^	Improvement in LVEF, wall motion score index, and perfusion of cardiac tissue
Menasché et al. (2008) [56]	33/34/30	SMB	4 × 10^8^/8 × 10^8^	No improvement in regional or global LVEF
Akar et al. (2009) [95]	25/25	ABMMNC	1.29 × 10^9^	Improvement in LVEF, perfusion, and contractility
Viswanathan et al. (2010) [96]	15/15	ABMMNC	3 × 10^6^–2.6 × 10^7^	Improvement in perfusion but no significant improvement in LVEF
Nasseri (2012) [88]	30/30	CD133 + BMC	5.6 × 10^6^	No improvement in LVEF

ABMMNC: autologous bone marrow mononuclear cells; BMC: bone marrow cells; LVEF: left ventricular ejection fraction; SMB: skeletal myoblast.

**Table 2:** Catheter-based transendocardial injection studies.

Study	No. of patients	Cell type	No. of cells	Outcome
Smits et al. (2003) [54]	5/0	SMB	2.96 × 10^8^	Improvement in LVEF
Perin et al. (2004) [97]	11/9	ABMMNC	2 × 10^6^	Improvement in exercise capacity and myocardial perfusion
Fuchs et al. (2006) [98]	27/0	BMC	2.8 × 10^7^	Improvement in perfusion and LVEF
Briguori et al. (2006) [99]	10/0	CD34 + /CD45 + BMC	4.6 × 10^6^	Improvement in myocardial perfusion
de la Fuente et al. (2007) [100]	10/0	ABMMNC	8.6 × 10^7^	Improvement in LVEF
Tse et al. (2007) [101]	9/10/9	BMC	1 × 10^6^/2 × 10^6^	Improvement in LVEF
Van Ramshorst et al. (2009) [102]	25/25	ABMMNC	1 × 10^8^	Modest improvement in myocardial perfusion
Trachtenberg (2011) [103]	40/20	MSC/BMC	2 × 10^8^	Minor improvement in LVEF
Williams et al. (2011) [104]	8/0	MSC/ABMMNC	7.63 × 10^8^	Improvement in regional contractility
Perin et al. (2012) [105]	61/31	BMC	1 × 10^8^	No improvement in LVEF
Perin et al. (2012) [105]	10/10	ALDH^br^	N/A	Improvement in cardiac function and perfusion of cardiac tissue

ABMMNC: autologous bone marrow mononuclear cells; ALDH^br^: aldehyde dehydrogenase bright cells; BMC: bone marrow cells; LVEF: left ventricular ejection fraction; MSC: mesenchymal stem cells; SMB: skeletal myoblasts.

**Table 3:** Intracoronary artery administration studies.

Study	No. of patients	Cell type	No. of cells	Outcome
Strauer et al. (2002) [102]	20	ABMMNC	2.8 × 10^7^	No significant LVEF improvement versus control
Chen et al. (2004) [135]	69	SMB	6 × 10^10^	Improvement in LVEF
Strauer et al. (2005) [136]	36	ABMMNC	9 × 10^7^	Improvement in LVEF
Erbs et al. (2005) [137]	26	CPC	7 × 10^7^	No significant LVEF improvement versus control
Bartunek et al. (2005) [138]	35	CD133 + BMC	1.3 × 10^7^	No significant LVEF improvement versus control
Katritsis et al. (2005) [139]	22	MSC/EPC	3 × 10^6^	No significant LVEF improvement versus control
Ruan et al. (2005) [140]	20	BMC	N/A	Improvement in LVEF
Assmus et al. (2006) [141]	51	ABMMNC	2 × 10^8^	Improvement in LVEF
Meluzín et al. (2006) [142]	66	ABMMNC	1 × 10^8^	Improvement in LVEF
Schächinger et al. (2006) [143]	204	ABMMNC	2.4 × 10^8^	Decreased mortality
Ge et al. (2006) [144]	20	ABMMNC	4 × 10^7^	No significant LVEF improvement versus control
Janssens et al. (2006) [145]	67	ABMMNC	1.7 × 10^8^	No significant LVEF improvement versus control
Lunde et al. (2006) [146]	100	ABMMNC	8.7 × 10^7^	Improvement in LVEF
Meyer et al. (2006) [147]	60	ABMMNC	2.5 × 10^9^	No significant LVEF improvement versus control
Assmus et al. (2006) [141]	47	CPC	2 × 10^7^	No significant LVEF improvement versus control
Schächinger et al. (2006) [143]	204	ABMMNC	2.4 × 10^8^	Improvement in LVEF
Won et al. (2006) [126]	82	CPC	1.4 × 10^9^	No significant LVEF improvement versus control
Li et al. (2007) [146]	70	CPC	7.3 × 10^7^	Improvement in LVEF
Chen et al. (2006) [148]	48	SMB	5 × 10^6^	No significant LVEF improvement versus control
Meluzín et al. (2008) [149]	60	ABMMNC	1 × 10^8^	Improvement in LVEF
Tatsumi et al. (2007) [150]	54	CPC	5 × 10^9^	Improvement in LVEF
Choi et al. (2007) [151]	73	CPC	2 × 10^9^	No significant LVEF improvement versus control
Herbots et al. (2009) [152]	33/34	BMC	N/A	Better recovery of LV function
Beitnes et al. (2009) [153]	50/50	BMC	6.8 × 10^7^	Improvement in exercise tolerance
Plewka et al. (2009) [154]	40/20	BMC	1.44 × 10^8^	Improvement in LV function
Tendera et al. (2009) [155]	80/40	BMC	1.78 × 10^8^	Longer delay between symptoms and revascularization
Strauer et al. (2010) [156]	191/200	BMC	6.6 × 10^7^	Improvement in LV function
Traverse et al. (2010) [157]	30/10	BMC	1 × 10^8^	Favorable effect on LV modeling
Quyyumi et al. (2011) [158]	16/15	CD34 + BMC	5, 10, and 15 × 10^6^	Dose dependent LV function improvement

ABMMNC: autologous bone marrow mononuclear cells; BMC: bone marrow cells; CPC: cardiac progenitor cells; EPC: endothelial progenitor cells; LVEF: left ventricular ejection fraction; MSC: mesenchymal stem cells; SMB: skeletal myoblasts; results combined from two reviews.
